# Genotoxic effects of association of the biogenic amines cadaverine and putrescine on the *Xenopus tropicalis* fibroblast cell line

**DOI:** 10.1007/s11356-026-38026-x

**Published:** 2026-07-17

**Authors:** Letícia Rocha Gonçalves, Letícia Tamborlin, Augusto Ducati Luchessi, Nicolas Pollet, Maria Aparecida Marin-Morales

**Affiliations:** 1 Department of General and Applied Biology, Institute of Biosciences, São Paulo State University Júlio de Mesquita Filho, Av. 24-A 1515, Rio Claro, SP 13506-900 Brazil; 2 Laboratório de Biotecnologia BraPhyto, Faculdade de Ciências Aplicadas (FCA), Universidade Estadual de Campinas (UNICAMP), Rua Pedro Zaccaria 1300, Limeira, SP 13484-419 Brazil; 3 UMR Évolution, Génomes, Comportement et Écologie, Université Paris-Saclay, CNRS, IRD, 91198 Gif-Sur-Yvette, France

**Keywords:** Cemetery pollution, DNA damage, Cell death, Necro-leachate, Amphibian cell line, Environmental impact

## Abstract

During cadaveric decomposition, necro-leachate is released into the surrounding environment, containing organic compounds such as the biogenic amines cadaverine (CAD) and putrescine (PUT). In this study, we performed the first integrated assessment of the genotoxic effects of CAD and PUT combinations at different concentrations (CAD: 0.3 × 10⁻^3^, 0.6 × 10⁻^3^, and 1.1 × 10⁻^3^ mol/L; PUT: 0.6 × 10⁻^3^, 6.0 × 10⁻⁶, and 6.0 × 10⁻⁷ mol/L) using *Xenopus tropicalis* (Speedy lineage) fibroblast cells. We evaluated genotoxicity via comet assay, micronucleus test, and flow cytometry. After 24 h of exposure, cells exposed to a combination of CAD 0.3 × 10⁻^3^ mol/L and PUT 6.0 × 10⁻⁶ mol/L (8) exhibited significantly increased frequency of micronuclei (1.88 vs. 0.88 in the negative control; *p* <0.05) and nuclear budding (3.11 vs. 0.70; *p* <0.05). We observed nuclear alterations (3.47 vs. 0.70; *p* <0.05) for CAD 0.3 × 10⁻^3^ mol/L + PUT 6.0 × 10⁻⁷ mol/L (9). After 48 h, the highest concentration tested CAD 1.1 × 10⁻^3^ + PUT 0.6 × 10⁻^3^ mol/L (5) induced marked DNA fragmentation (~ 9% vs. 1% in the negative control; *p < *0.05), S-phase cell cycle arrest (~ 12% vs. 7% in the negative control; *p* <0.05) and apoptosis. These findings confirm the genotoxic potential of 8 and 9 and whereas 5 was associated with more pronounced apoptotic and cytotoxic effects. Our results provide mechanistic evidence of the genotoxic potential of CAD and PUT combinations, supporting further investigation of their environmental and toxicological relevance.

## Introduction

Environmental degradation results from human activities that alter the physical and chemical conditions of the environment, making anthropogenic impacts one of the greatest challenges of the twenty-first century (Saba et al. 2023). Cemeteries are among the primary anthropogenic sources of environmental contamination (Jonker and Olivier 2012). Necropolises are considered a major source of soil and surface water pollution on a global scale due to the high concentration of corpses, which release physical, chemical, and biological contaminants into the environment, primarily as a result of the cadaveric decomposition process (Neckel et al. 2021; Azevedo et al. 2023).

The primary source of environmental contamination in cemeteries is necro-leachate, a liquid released during cadaveric putrefaction. A 70-kg body can generate approximately 45 L of this substance, which consists of 60% water, 30% mineralized compounds and 10% organic compounds. Organic compounds, including toxic biogenic amines such as cadaverine (CAD) and putrescine (PUT), are present in necro-leachate (Gonçalves et al. 2021 and Oliveira et al. 2013).

Both amines are small molecules, produced by the decarboxylation of amino acids through enzymatic processes (Bachrach 2004). They are present in the body in micromolar concentrations and participate in some cellular processes such as transcription, translation, growth, division, and cell death (Bach et al. 2012 and Ramani et al. 2014). These amines, due to their nucleophilic sites, can interact with macromolecules such as nucleic acids, lipids, and proteins (Hussain et al. 2011).

At concentrations above the physiological levels, CAD and PUT amines can interfere with the stability of the macromolecules, promoting DNA/protein damage (Unal et al. 2008 and Hussain et al. 2011). They can also generate oxidative stress events (Alzoghaibi 2013). These events can cause DNA damage and alter gene transcription and protein translation processes, changes that may lead to genomic instability or cell death. Thus, the impacts of the presence of CAD and PUT are strongly associated with the concentration of amines in the cells, as well as the tissue involved (Bekebrede et al. 2020 and Gonçalves et al. 2021).

Cadaverine and putrescine coexist in necro-leachate and are therefore simultaneously present in environmental exposures. Although precise environmental concentrations are not available, their concurrent occurrence justifies assessing combined effects. Studies in plants and aquatic organisms indicate that simultaneous exposure can potentiate cytotoxic and mutagenic effects compared to single-compound treatments (Braga et al. 2024a, b), suggesting non-additive interactions. Mechanistically, as positively charged diamines, CAD and PUT can interact with negatively charged cellular components such as DNA and membranes (Unal et al. 2008; Del Rio et al. 2019). Because intracellular polyamine homeostasis tightly regulates chromatin organization, gene expression, and cell cycle progression (Igarashi and Kashiwagi 2010; Pegg 2016), simultaneous exposure may perturb this balance differently from individual exposures, providing a plausible mechanistic rationale for studying their mixture.

The ecotoxicological effects of biogenic amines present in necro-leachate have been investigated in various organisms and cell cultures. Braga et al. (2024a) reported that combined exposure to CAD and PUT is phytotoxic to *Lactuca sativa* (lettuce) and *Allium cepa* (onion).

In a separate study, Braga et al. (2024b) confirmed the toxic effects of the CAD and PUT mixture on aquatic organisms, including *Danio rerio* (zebrafish) and *Daphnia magna* (water flea). They observed delayed hatching and malformations during embryonic development in fish, as well as increased lethality in *D. magna*. Additionally, research on soil organisms, specifically *Folsomia candida* (springtails), indicated a preference for soil treated with biogenic amines, as shown by avoidance tests. However, chronic exposure to CAD and PUT adversely affected their reproductive activity, primarily due to high adult mortality rates (Braga et al. 2023).

Campos-Pereira et al. (2025) investigated the effects of various concentrations of putrescine on human hepatoma cell cultures (HepG2), demonstrating that putrescine induces genotoxic effects and alters gene expression in oxidative stress and DNA damage pathways. Del Rio et al. (2019) reported that exogenous concentrations of cadaverine and putrescine caused cytotoxicity in human intestinal cell cultures (HT29). Chen et al. (2015) found that putrescine affected human skin fibroblast (HSF) cell lines by inducing either cell proliferation or apoptosis, depending on its concentration.

In vitro assays using cell lines are valuable tools for evaluating the effects of various agents on vertebrate systems. Moreover, these tests serve as effective alternatives to the principles of replacement, reduction, and refinement (3Rs) in animal experimentation, promoting animal welfare and ethical research practices (Araújo et al. 2014; Quadros et al. 2020; Vichare et al. 2021). Studies about cemetery contaminants in vitro systems of vertebrates have only been conducted using human cell lines.

The use of a non-mammalian vertebrate system broadens the taxonomic scope of toxicity assessment in studies involving environmentally derived contaminants such as necro-leachate, overcoming the current limitation of investigations restricted to human cell lines. While fish and mammalian cell lines are commonly employed in ecotoxicological studies, they represent either exclusively aquatic systems or strictly mammalian physiology. Amphibians, by contrast, occupy an intermediate evolutionary position and experience both aquatic and terrestrial exposure scenarios (Brühl et al. 2013), making them particularly relevant for assessing environmentally derived contaminants such as necro-leachate.

Amphibians are widely recognized as sensitive bioindicators of environmental contamination due to their highly permeable skin and exposure across aquatic and terrestrial habitats, which increase their susceptibility to pollutants (Brühl et al. 2013). The Speedy cell line is derived from the fibroblasts of the amphibian *X. tropicalis* and it has a chromosome number of 2n = 21, with trisomy on chromosome 10 (Sinzelle et al. 2012). The genome of this species exhibits regions of genetic collinearity with humans. Among the more than 20,000 genes of *X. tropicalis*, 1700 are orthologous to human disease-related genes, and over one-third of its genome consists of transposable elements, with a density 40% to 50% comparable to that observed in mammals (Hellsten et al. 2010). This genomic conservation supports the mechanistic relevance of this model, enabling the identification of fundamental genotoxic and cell cycle–related effects that are highly conserved across vertebrates (Hellsten et al. 2010; Ciccia and Elledge 2010). *X. tropicalis* has become a valuable model organism for genetic, biological, and biomedical research (Bowes et al. 2008; Zhu et al. 2019).

Despite these advances, important knowledge gaps remain. Although the toxicological effects of cadaverine and putrescine have been investigated individually and, in some cases, in combination in plant and invertebrate models, their combined genotoxic effects have not yet been evaluated in vertebrate in vitro systems. Furthermore, vertebrate cell-based studies addressing cemetery-related contaminants have been restricted to human cell lines, limiting ecological relevance and comparative extrapolation. Considering that CAD and PUT coexist in necro-leachate, evaluating their combined cellular effects in a non-mammalian vertebrate model represents a critical step toward clarifying their mechanistic and ecotoxicological impact.

Given the toxicity of non-physiological concentrations of the biogenic amines CAD and PUT and their documented presence in cemetery environments, this study examined their genotoxic effects by assessing DNA damage and cell cycle alterations in Speedy cells. These findings provide baseline data that contribute to the understanding of their broader ecotoxicological potential, while highlighting the use of this cell line as a pioneering model for environmental research.

## Material and methods

### Tested substances

Associations of different concentrations of the biogenic amines cadaverine (CAD,1.5-pentanediamine, NH2(CH2)_5_NH_2_, molecular weight of 102.18, CAS: 462–94-2, purity of 97%) and putrescine (PUT, 1.4-butanediamine, C4H12N2, molecular weight of 88.15 g/mol, CAS No. 110–60-1, purity of 99.0%) were used. Both substances were purchased from Sigma-Aldrich, Barueri, SP, Brazil.

There is no available data in the literature regarding the LD50 of CAD, nor the LD50 of CAD and PUT for amphibians. Therefore, the concentrations selected for this study were based on the LD50 concentration of putrescine for rats (463 mg/kg, according to the product’s technical information), as an alternative strategy frequently employed in toxicological studies when species-specific data are unavailable (Campos-Pereira et al. 2025). Subsequently, serial dilutions were prepared in Leibowitz L-15 medium, and Table 1 presents the PUT concentrations utilized in this study.

**Table 1:** Associations of different concentrations of cadaverine (CAD) and putrescine (PUT) tested in Speedy cells

Association	Cadaverine – mol/L	Putrescine – mol/L
1	1.1 × 10^−3^	6.0 × 10^−7^
2	1.1 × 10^−3^	6.0 × 10^−6^
3	0.3 × 10^−3^	0.6 × 10^−3^
4	0.6 × 10^−3^	0.6 × 10^−3^
5	1.1 × 10^−3^	0.6 × 10^−3^
6	0.6 × 10^−3^	6.0 × 10^−6^
7	0.6 × 10^−3^	6.0 × 10^−7^
8	0.3 × 10^−3^	6.0 × 10^−6^
9	0.3 × 10^−3^	6.0 × 10^−7^

### Biological material

The cells derived from amphibian fibroblasts (Speedy) were used as a study model for the Resazurin toxicity assays; genotoxicity with comet and MN assays with cytokinesis blocks; and cell cycle alteration and nuclear fragmentation with flow cytometry. The cell line used was provided by Prof. Dr. Nicolas Pollet from the Institute of Systems and Synthetic Biology (iSSB), Genopole, France and was inserted into the cell bank of the Environmental Mutagenesis Laboratory of the Department of General and Applied Biology, Institute of Biosciences, UNESP, Rio Claro, SP, Brazil.

Cell cultures were performed using a Leibovitz L-15 medium, prepared with an antibiotic/antimycotic solution, and supplemented with 10% fetal bovine serum. Unventilated 25 cm^2^ flasks were used in a BOD incubator at 28 °C. Under these conditions, the doubling time was 24 h. Cells used in the experiments ranged between passages 11 and 17. Cell morphology and growth characteristics were routinely monitored and remained consistent with the original description of the Speedy cell line (Sinzelle et al. 2012). Furthermore, the stability and health of the cultures were confirmed by consistent growth kinetics and the low basal DNA fragmentation (sub-G0/G1 population) observed in negative control groups during flow cytometry analysis (as detailed in the “Results” section). These parameters are recognized as sensitive indicators of the absence of covert contamination (Drexler and Uphoff 2002; Geraghty et al. 2014). Cultures were maintained under strict aseptic conditions throughout the experimental period. Mycoplasma was not detected in Speedy cell lines using qPCR as described by Siegl et al. (2023), with the primers MYCOF (5′-TYCTACGGGAGGCAGCAG-3′) and MYCOR (5′-CGRCTGCTGGCACATAGTT-3′). The qPCR assays were performed using 5 µL of cell culture supernatant obtained from independently grown cell cultures.

### Cell viability assay

For the resazurin assay with the Speedy cell line, the cells were exposed to the nine concentrations of the combination of CAD and PUT described in Table 1 and according to the protocol by O`Brien et al. (2000), with some modifications. For that, 2.34 × 10^4^ Speedy cells were seeded in 96-well plates and incubated for 24 h in an incubator at 28 °C. Then, the culture medium was replaced with a fresh medium containing different concentrations of CAD and PUT (Table 1). The negative control (NC) was prepared with cell culture medium and the positive control (PC) with cell culture medium and 1% Triton X-100 (Borner et al. 1994). After 24 h, the treatments were removed and a resazurin solution (44 µM) with cell culture medium was added to each well. After 4 h of incubation, the results were measured in an Infinite M200 Pro™ model plate reader (Tecan, Crailsheim, Germany), at 590 nm. The assay was performed in triplicate. Cellular viability below 80% was considered cytotoxic (ISO 10993–5:2009 2009; López-García et al. 2014). Then, three non-cytotoxic concentrations of the association of CAD and PUT were selected to be used in comet assay, cytokinesis blocking MN test and flow cytometry analyses; association number five of biogenic amines (Table 1) was also utilized for flow cytometry analyses (Table 2).

**Table 2:** Associations of cadaverine (CAD) and putrescine (PUT) selected for subsequent biological assays after cell viability screening

Association	Cadaverine	Putrescine	Assays
	mol/L	mol/L	
6	0.6 × 10^−3^	6.0 × 10^−6^	Comet, MN and Flow cytometry
8	0.3 × 10^−3^	6.0 × 10^−6^	Comet, MN and Flow cytometry
9	0.3 × 10^−3^	6.0 × 10^−7^	Comet, MN and Flow cytometry
5	1.1 × 10^−3^	0.6 × 10^−3^	Flow cytometry

### Comet assay

The alkaline comet assay was performed according to Campos-Pereira et al. (2025), with some modifications. For this, 1 × 10^6^ Speedy cells were grown in individual flasks to promote cell culture stabilization, and incubated for 24 h in an incubator at 28 °C. Then, the culture medium was replaced with a fresh medium containing three different concentrations of the association of CAD and PUT (Table 2), and incubated for 24 h in an incubator at 28 °C. The NC consisted of a cell culture medium, and the PC consisted of a 10 mM methylmethane sulfonate (MMS) solution, prepared by diluting 3.33 μL of MMS (Sigma-Aldrich) in 4 mL of PBS. Subsequently, 50 μL of this solution was added to 5 mL of specific medium in the positive control flasks.

Before mounting the cell suspension slides, the cell viability test was performed using Trypan Blue (0.4%), quantifying in a Neubauer chamber the presence of white (viable cells) and blue (non-viable cells). When cell viability was equal to or > 80%, the experiment was continued.

Two slides covered with common agarose were mounted for each flask with 20 μL cell suspension plus 120 μL low-melting-point agarose at 37 °C. Subsequently, they were subjected to lysis solution (1 mL Triton X-100, 10 mL DMSO, and 89 mL stock lysis solution: 2.5 M NaCl, 100 mM EDTA, 10 mM Tris, pH 10, about 8 g solid NaOH to 1 L), they were kept at pH 10 and in the dark at 4 °C for 1 h. Then, the electrophoretic run was performed for 20 min in a buffer (300 mM NaOH + 1 mM EDTA, with pH > 13) at 39 V and 300 mA (~ 0.8 V/cm). Afterwards, the slides were neutralized with buffer (pH 7.2) and fixed for 10 min in absolute ethanol. The slides were stained with 50 μL GelRed® solution (15 μL 10,000× GelRed in water, 5 mL 1 M NaCl, and 45 mL distilled water) (Reisinger et al. 2018) and analyzed in a Leica fluorescence microscope, filter B–34 (excitation: i = 420–490 nm, barrier: i = 520 nm). The test was carried out in triplicate and the count was done at random, with 40 × objective. DNA damage was assessed in 100 nucleoids per slide, and 600 nucleoids per treatment, and slides were selected and scored randomly and blindly to treatment to minimize potential bias. In this assay, the parameters analyzed were DNA intensity using Comet Assay IV software (Perspective Instruments, Bury Saint Edmunds, UK), and no categorical scoring thresholds were applied, as the software provides objective, continuous measurements for each nucleus (Collins et al. 2023). No cytostasis correction was performed, as only concentrations that maintained cell viability ≥ 80%, confirmed by Trypan Blue staining prior to the comet assay, were used; these are considered non-cytotoxic. For the statistical analyses, the normality and homogeneity were performed using the Shapiro–Wilk and Levene tests, respectively; followed by the Kruskal–Wallis and Dunn tests. The significance level used in the hypothesis tests was *p* < 0.05.

### Cytokinesis blocking micronucleus test (CBMN)

The CBMN test was performed according to the protocol by Natarajan and Darroudi (1991), with some modifications. 1 × 10^6^ Speedy cells were cultivated in 25 cm^2^ flasks, and incubated for 24 h in an incubator at 28 °C. The cells were exposed to 3 different concentrations of the association of CAD and PUT, described in Table 2, for a period of 24 h. The NC consisted of a cell culture medium, and the PC consisted of a 10 mM MMS (Sigma-Aldrich) solution, prepared by diluting 3.33 μL of MMS in 4 mL of PBS. Subsequently, 50 μL of this solution was added to 5 mL of specific medium in the positive control flasks. The experiment was carried out in triplicate.

After exposure, the cells were washed with PBS and 5 mL of complete culture medium containing 3 µg/mL of cytochalasin B. The working solution was prepared from the stock solution of cytochalasin B, Sigma® (2 mg/mL: 5 mg of cytochalasin B, dissolved in 2.5 mL of dimethyl sulfoxide, Exôdo Científica®) by diluting 50 µL of the stock solution in 1.65 mL of sterile PBS, and stored according to the manufacturer’s instructions. The flasks were incubated as described in item 2.3, for 28 h (Tsuboy et al. 2007 and Chequer et al. 2009). Cells were harvested, fixed with Carnoy's solution. Slides were prepared with these cells and subjected to hydrolysis with 1 N HCl for 10 min at 60 °C. Then, the slides were exposed for 2 h to Shiff’s reagent, and then washed with running water. After drying, the slides were stained with 5% Giemsa for 8 min. The slides were analyzed under a light microscope at 1000× magnification. The assay was performed in triplicate. 2000 cells were counted per replicate, totaling 6000 cells for each concentration and the controls.

For the evaluation of the genotoxicity of the combinations of CAD and PUT, nucleoplasmic bridges and/or nuclear buds were considered, and for mutagenic potential, binucleated cells carrying MN (Fenech et al. 2003) were examined. The evaluation of the cell division index (CDI) was performed by counting 1000 cells/treatment. Then, IDC was calculated (Eastmond and Tucker 1989). For the statistical analyses, the normality and homogeneity were assessed using the Shapiro–Wilk and Levene tests, respectively; then ANOVA followed by a post hoc Dunnett test was performed. The significance level used in the hypothesis tests was *p* < 0.05.

### Cell cycle profile and DNA fragmentation by flow cytometry

The cell cycle distribution and the percentage of cells with fragmented DNA were evaluated using the results obtained from measurements of the DNA content, after labeling with propidium iodide (PI). 2.5 × 10^5^ cells were cultivated in 6-well plates, and incubated for 24 h in an incubator at 28 °C. Initially, the cells were exposed over a period of 24 h to 3 different associations (6, 8, and 9; Table 2). As no alterations in the cell cycle and DNA fragmentation were observed for these associations, an additional association was also tested, with a higher concentration of amines (CAD 1.1 × 10^–3^ mol/L + PUT 0.6 × 10^–3^ mol/L, shown in Table 1 as Association 5). A new test was then carried out with these 4 associations at 24 and 48 h of exposure (Table 2). The NC was performed with a cell culture medium, and the PC consisted of a 10 mM MMS (Sigma-Aldrich) solution, prepared by diluting 3.33 μL of MMS in 4 mL of PBS. Subsequently, 50 μL of this solution was added to 5 mL of specific medium in the positive control flasks.

After treatment, cells were collected by trypsinization and transferred to microcentrifuge tubes. The tubes were centrifuged at 300 g for 5 min at room temperature. Next, the cells were washed with PBS. Cells were then fixed with 70% ethanol in an ice bath for 30 min. Subsequently, the cells were centrifuged at 300 × g for 5 min and washed with PBS. Then, 200 µL of staining solution (20 µg/mL PI), 10 µg/mL RNase A, and 0.1% Triton X-100 were added to the cells, where they were incubated in the dark at room temperature for 30 min. The cells were analyzed using a BD Accuri™ C6 flow cytometer (BD Biosciences, San Jose, CA, USA). 10,000 events per sample were recorded using the FL-2A channel to determine the DNA content of the cells.

For the analysis, the gating strategy involved an initial FSC-A vs. SSC-A plot to exclude debris, followed by pulse processing (FSC-A vs. FSC-H) to discriminate doublets and ensure the analysis of single cells. The sub-G0/G1 population was quantified and interpreted as an indicator of DNA fragmentation associated with apoptotic processes (Riccardi and Nicoletti 2006). The percentages of cells in the different phases of the cell cycle were configured from the collected data, using the BD Accuri™ C6 software (BD Biosciences, San Jose, CA, USA). For the statistical analyses, three independent experiments were carried out with cells from different batches. All experiments were performed in triplicate. The test results were submitted to statistical analyses. Values are presented as mean ± standard error (SEM). Differences between treatments were addressed using one-Way ANOVA, followed by Tukey’s post hoc test. Differences were considered statistically significant at *p* < 0.05.

## Results

In the present study, assays were conducted using the amphibian fibroblast cell line (Speedy), which provided data that contribute to a better understanding of the biological effects of the two most significant components of cemetery leachate: cadaverine (CAD) and putrescine (PUT). Through the resazurin assay, it was possible to select the concentrations of the CAD and PUT combination that were non-cytotoxic to the Speedy cell line for use in genotoxicity and flow cytometry assays. Associations 6, 8, and 9 resulted in cell viability between 80 and 100% (ISO 10993–5:2009 2009; López-García et al. 2014) (Table 3). These associations were used in comet assays, MN assays with cytokinesis blocking, and flow cytometry analyses (Table 2).

**Table 3:** Results of the cell viability test (Resazurin) on Speedy cells exposed to different associations of cadaverine (CAD) and putrescine (PUT)

Association	Cadaverine mol/L	Putrescine mol/L	Cell viability Mean ± SD
1	1.1 × 10^–3^	6.0 × 10^–7^	137.21 ± 6.91%
2	1.1 × 10^–3^	6.0 × 10^–6^	153.13 ± 12.59%
3	0.3 × 10^–3^	0.6 × 10^–3^	157.98 ± 5.14%
4	0.6 × 10^–3^	0.6 × 10^–3^	161.55 ± 10.37%
5	1.1 × 10^–3^	0.6 × 10^–3^	152.44 ± 12.72%
6	0.6 × 10^–3^	6.0 × 10^–6^	80.16 ± 12.67%
7	0.6 × 10^–3^	6.0 × 10^–7^	75.67 ± 5.83%
8	0.3 × 10^–3^	6.0 × 10^–6^	82.51 ± 5.76%
9	0.3 × 10^–3^	6.0 × 10^–7^	84.25 ± 4.17%

**Fig. 1:**
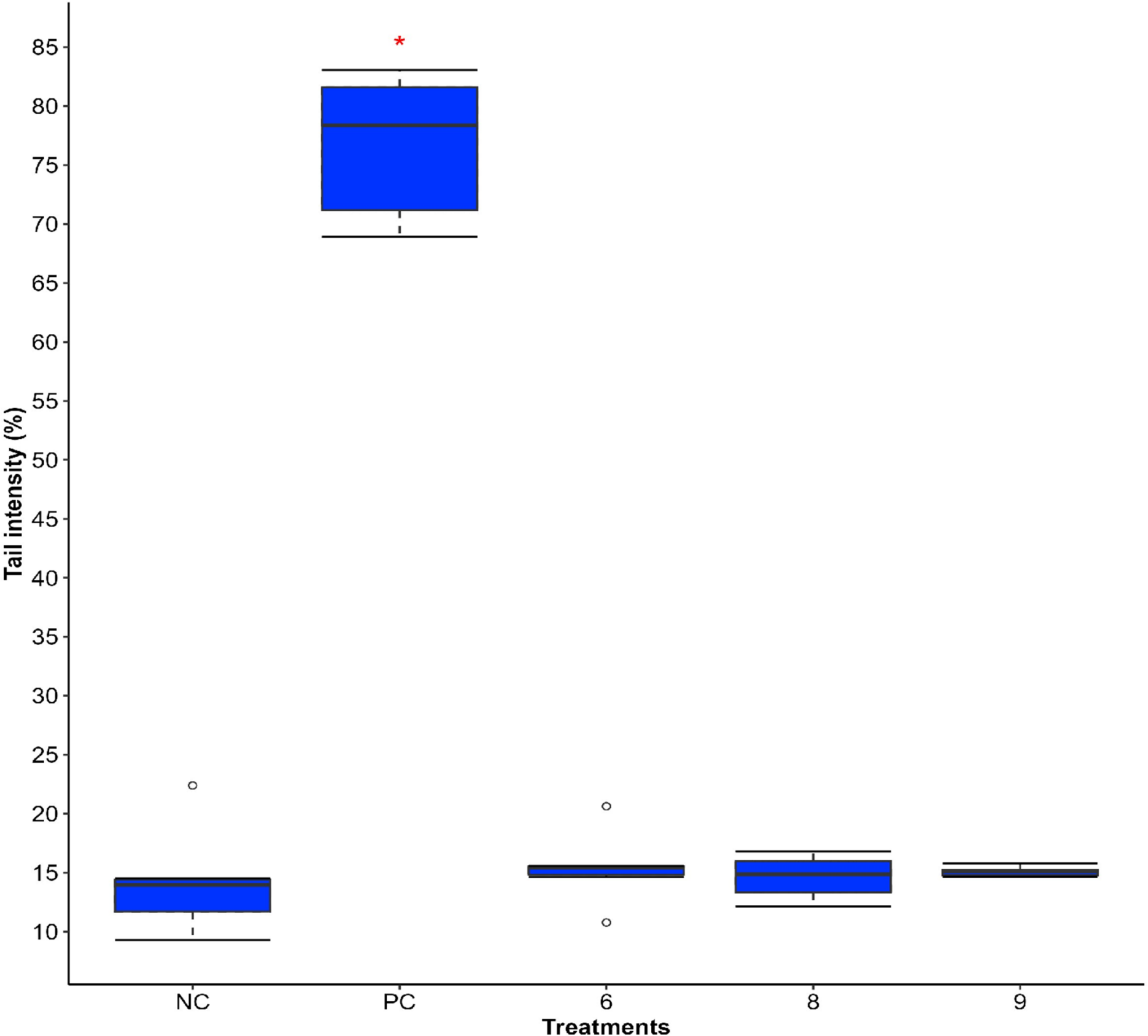
DNA percentage in the tail of Speedy cells exposed to treatments with different combinations of cadaverine (CAD) and putrescine (PUT). NC: negative control; PC: positive control; 6 = 0.6 × 10^−3^ mol/L CAD + 6.0 × 10^−6^ mol/L PUT; 8 = 0.3 × 10^−3^ mol/L CAD + 6.0 × 10^−6^ mol/L PUT; 9 = 0.3 × 10^−3^ mol/L CAD + 6.0 × 10^−7^ mol/L PUT. * Statistically different in relation to NC (Kruskal–Wallis/Dunn’s test, *p* < 0.05)

Regarding the Comet assay, the results obtained by the tail intensity criterion showed that there was no statistically significant difference between the tested treatments (6, 8, and 9) and the NC (Fig. 1). These results make it possible to infer that these associations of the biogenic amines CAD and PUT did not present genotoxic potential to the Speedy cells.

The cytotoxicity of Associations 6, 8, and 9 was evaluated using the CBMN assay, with a focus on the Cytokinesis Block Proliferation Index (CBPI). In the Speedy cells, no statistically significant differences were observed among the tested associations (6, 8, and 9) compared to the negative control (NC).

The genotoxicity of these associations in Speedy cells was assessed through the CBMN test, which measured the frequency of micronuclei (MN) and the formation of nuclear buds and bridges in binucleated cells.

According to the results of the micronucleus (MN) assay, Association 6 did not induce statistically significant genotoxic effects in Speedy cells under the experimental conditions tested. In contrast, the Association 8 promoted a statistically significant increase in micronucleus frequency compared to the negative control (NC). Additionally, Associations 8 and 9 significantly increased the frequency of nuclear buds relative to NC (Table 4). No statistically significant differences were observed in the frequency of nuclear bridges among 6, 8, or 9 when compared to NC. Overall, these findings indicate that Associations 8 and 9 exert genotoxic effects.

**Table 4:** Results of the MN assay on Speedy cells exposed to various associations of cadaverine (CAD) and putrescine (PUT)

	CBPI	MN	Buds	Bridges
	Mean ± SD	Mean ± SD	Mean ± SD	Mean ± SD
NC	1.74 ± 0.06	0.88 ± 0.43	0.70 ± 0.30	0.05 ± 0.05
PC	1.59 ± 0.03*	2.06 ± 0.03*	2.92 ± 0.46*	0.18 ± 0.10
6	1.80 ± 0.02	1.05 ± 0.10	0.98 ± 0.03	0.15 ± 0.17
8	1.75 ± 0.01	1.88 ± 0.32*	3.11 ± 0.29*	0.08 ± 0.08
9	1.69 ± 0.03	1.39 ± 0.38	3.47 ± 0.08*	0.08 ± 0.08

Values are expressed as mean ± standard deviation (SD) of the frequency of micronuclei (MN), nuclear buds, and nuclear bridges in Speedy cells exposed to different associations of cadaverine (CAD) and putrescine (PUT). *NC* negative control, *PC* positive control. 6 = 0.6 × 10^−3^ mol/L CAD + 6.0 × 10^−6^ mol/L PUT, 8 = 0.3 × 10^−3^ mol/L CAD + 6.0 × 10^−6^ mol/L PUT; 9 = 0.3 × 10^−3^ mol/L CAD + 6.0 × 10^−7^ mol/L PUT. A total of 2000 binucleated cells were scored per replicate (6000 cells per treatment; *n* = 3 independent experiments). Normality and homogeneity were assessed using the Shapiro–Wilk and Levene tests, respectively. Statistical analysis was performed using one-way ANOVA followed by Dunnett’s post hoc test. *p < 0.05 compared to NC

**Fig. 2:**
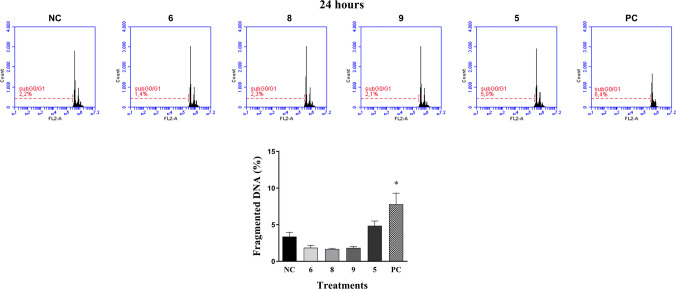
Analysis of DNA fragmentation by PI staining, evaluated by flow cytometry after treatment with associated cadaverine (CAD) and putrescine (PUT) on Speedy cells (sub-G0/G1 cell population). NC: negative control; 6 = 0.6 × 10^−3^ mol/L of CAD + 6.0 × 10^−6^ mol/L of PUT; 8 = 0.3 × 10^−3^ mol/L of CAD + 6.0 × 10^−6^ mol/L of PUT; 9 = 0.3 × 10^−3^ mol/L of CAD + 6.0 × 10^−7^ mol/L of PUT; 5 = 1.1 × 10^−3^ mol/L of CAD + 0.6 × 10^−3^ mol/L of PUT; PC: positive control

**Fig. 3:**
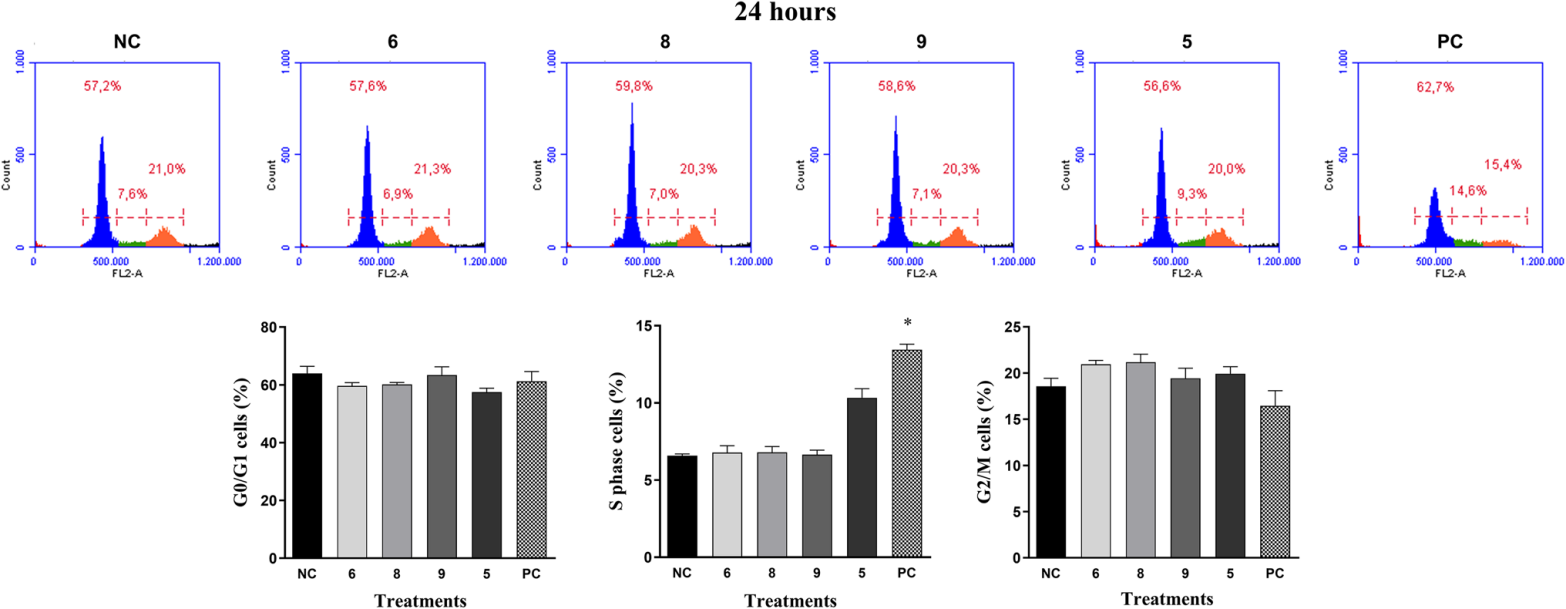
Cell cycle analysis (PI staining) by flow cytometry after treatment with associated cadaverine (CAD) and putrescine (PUT) in Speedy cells. NC: negative control; 6 = 0.6 × 10^−3^ mol/L of CAD + 6.0 × 10^−6^ mol/L of PUT; 8 = 0.3 × 10^−3^ mol/L of CAD + 6.0 × 10^−6^ mol/L of PUT; 9 = 0.3 × 10^−3^ mol/L of CAD + 6.0 × 10^−7^ mol/L of PUT; 5 = 1.1 × 10^−3^ mol/L of CAD + 0.6 × 10^−3^ mol/L of PUT; PC: positive control

Analysis conducted on the flow cytometer revealed the effects on cell cycle progression and cytotoxicity in Speedy cells exposed to different CAD and PUT associations for 24 and 48 h. The results obtained from the 24-h treatment assays showed no statistically significant differences in DNA fragmentation and cell cycle progression compared to the NC for any of the associations tested (Figs. 2 and 3).

**Fig. 4:**
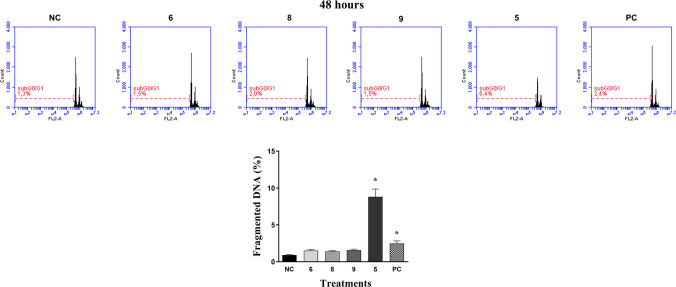
Analysis of DNA fragmentation by PI staining, evaluated by flow cytometry after treatment with associated cadaverine (CAD) and putrescine (PUT) in Speedy cells (sub-G0/G1 cell population). NC: negative control; 6 = 0.6 × 10^−3^ mol/L of CAD + 6.0 × 10^−6^ mol/L of PUT; 8 = 0.3 × 10^−3^ mol/L of CAD + 6.0 × 10^−6^ mol/L of PUT; 9 = 0.3 × 10^−3^ mol/L of CAD + 6.0 × 10^−7^ mol/L of PUT; 5 = 1.1 × 10^−3^ mol/L of CAD + 0.6 × 10^−3^ mol/L of PUT; PC: positive control. * Statistically different in relation to NC (Dunn’s test, *p* < 0.05)

**Fig. 5:**
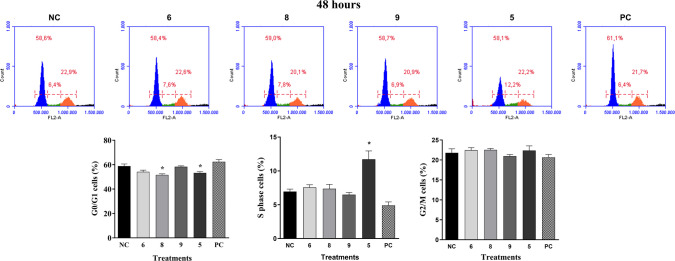
Cell cycle analysis (PI staining) by flow cytometry after treatment with associated cadaverine (CAD) and putrescine (PUT) in Speedy cells. NC: negative control; 6 = 0.6 × 10^−3^ mol/L of CAD + 6.0 × 10^−6^ mol/L of PUT; 8 = 0.3 × 10^−3^ mol/L of CAD + 6.0 × 10^−6^ mol/L of PUT; 9 = 0.3 × 10^−3^ mol/L of CAD + 6.0 × 10^−7^ mol/L of PUT; 5 = 1.1 × 10^−3^ mol/L of CAD + 0.6 × 10^−3^ mol/L of PUT; PC: positive control. * Statistically different in relation to NC (Dunn’s test, *p* < 0.05)

Assays performed with the same cell line submitted to 48-h treatments showed that Associations 6, 8, and 9 did not induce significant changes in DNA fragmentation compared to the control, but there was a significant increase (9%) in the percentage of cells with fragmented DNA for Association 5 (Fig. 4). It was also observed that the combination in Association 5 induced a cell cycle arrest in the phase that controls DNA duplication (S phase) after 48 h of treatment (Fig. 5). Association 5 significantly altered cell cycle progression, increasing the percentage of cells in the S phase from about 7.8% (Association 8) to 12.2% (Association 5) compared to the NC (Fig. 5).

Thus, 24 h of exposure to CAD and PUT associations in Speedy cells may not have been sufficient to induce deleterious effects for this study model.

## Discussion

From a scientific standpoint, there is a significant lack of public awareness regarding the environmental impacts of cadaveric decomposition (Campos-Pereira et al. 2025). Although the toxicity and potential health risks of the biogenic amines cadaverine (CAD) and putrescine (PUT) have been investigated due to their presence in food products (EFSA 2011; Ekici and Omer 2020; Świder et al. 2020; Muñoz-Esparza 2021), their environmental implications, particularly in areas surrounding cemeteries, remain poorly understood. The potential environmental risks associated with biogenic amines are of particular concern due to their water solubility, which facilitates their dispersion into various environmental compartments such as groundwater, surface water, and sediments (Gonçalves et al. 2021; Honscha et al. 2021). These amines, generated during the decomposition of organic matter, may thus contribute to broader ecological contamination. In this context, the present study introduces an innovative cell line model (Speedy) for environmental toxicological assessment, offering novel insights into the effects of necro-leachate constituents, such as biogenic amines, on both aquatic and terrestrial organisms. Furthermore, the bioassays conducted here may provide a preliminary basis for discussing the potential risks these compounds pose to human health, especially regarding groundwater quality.

Regarding cell viability assessment, some associations induced apparent hyper-viability (> 100%) in the resazurin assay. As reported by O’Brien et al. (2000), resazurin reduction depends on cellular reducing activity and may be influenced by the metabolic state of the cells, where accumulation of the fluorescent product may lead to an overestimation of the cell population. Therefore, the increased fluorescence observed here likely reflects enhanced cellular reducing capacity rather than a direct increase in cell number. Importantly, these specific concentrations were not selected for subsequent genotoxicity assays. Only concentrations maintaining at least 80% cell viability were used, following ISO 10993–5:2009 (2009) (López-García et al. 2014), thereby minimizing potential confounding effects on downstream genotoxicity endpoints.

This study evaluated the genotoxicity of various non-cytotoxic combinations of cadaverine (CAD) and putrescine (PUT) (Associations 6, 8, and 9) in fibroblast cells (Speedy) using a comprehensive panel of bioassays. These assays are highly sensitive and widely applied for detecting primary DNA damage, including single and double-strand breaks, alkali-labile sites, and DNA-DNA or DNA-protein crosslinks. For regulatory purposes, the most widely accepted parameter is the percentage of DNA in the tail, which reflects the extent of DNA fragmentation (Olive 1999; Collins et al. 2008; Kumaravel et al. 2009). Results from the present study demonstrate that the tested combinations of cadaverine (CAD) and putrescine (PUT) (Associations 6, 8 and 9) did not induce DNA strand breaks.

Therefore, based on the percentage of DNA in the tail measured after 24 h of exposure, no detectable DNA strand breaks were observed in the comet assay. However, it is well established that primary DNA lesions, particularly single-strand breaks, are short-lived and may be detectable only within a limited time window between 2 and 6 h following exposure (Brendler-Schwaab et al. 2005). For this reason, additional genotoxicity endpoints were assessed using the cytokinesis-block micronucleus (CBMN) assay.

In the present study, we evaluated the CBPI, the presence of buds, and nuclear bridges, in addition to performing the MN test. The first two indicators were employed to assess genotoxicity, while the MN test was used to evaluate mutagenic potential (Fenech 2000; OECD 2014; OECD 2017; Ren et al. 2017; Verheyen et al. 2017). In the CBPI assay conducted in Speedy cells, Associations 6, 8, and 9 did not exhibit a significant increase in proliferative activity compared to the negative control group.

Regarding the parameters of genotoxicity (nuclear buds) and mutagenicity potential (MN), no genotoxic effects were observed for the association of the highest concentrations of CAD and PUT (6). However, assays involving associations of lower concentrations of biogenic amines revealed that Association 8 induced genotoxic and potentially mutagenic effects, whereas 9 exhibited genotoxicity in Speedy cells. None of the tested combinations (6, 8, or 9) resulted in the formation of internuclear bridges.

Micronuclei (MNs), nuclear buds, and nucleoplasmic bridges are cytogenetic endpoints extensively employed in genotoxicity assays to detect chromosomal damage and mitotic disturbances induced by xenobiotics (Fenech et al. 2011). MNs originate from acentric chromosomal fragments or whole chromosomes that fail to properly segregate and are excluded from the primary nucleus during anaphase-telophase, resulting from aneugenic or clastogenic mechanisms of the test compound (Sommer et al. 2020; Heaven et al. 2022). Nuclear buds, observed at the nuclear periphery, represent amplified or excess DNA that is extruded from the nucleus during S-phase due to gene amplification events or elimination of damaged DNA. This process can be associated with aneugenic activity, as it involves perturbations in spindle fiber dynamics and kinetochore function, ultimately leading to chromosomal missegregation and polyploidization (Fenech and Crott 2002 and Fenech et al. 2011). Nucleoplasmic bridges, conversely, arise from dicentric chromosomes or misrepaired DNA double-strand breaks and are indicative of clastogenic effects, reflecting chromosomal rearrangements and replication stress (Fenech 2006). These biomarkers, routinely evaluated using the cytokinesis-block micronucleus (CBMN) assay, provide an integrated measure of chromosomal instability and are considered robust indicators of genotoxic potential in eukaryotic cells.

In the micronucleus (MN) assays conducted with the Speedy cell line, genotoxic alterations were detected in both Associations 8 and 9 samples, alongside evidence of mutagenicity potential in Association 8, pointing toward a potential aneugenic mode of action associated with these treatments. This interpretation is further supported by the absence of nucleoplasmic bridges in the MN assay.

The results of the micronucleus (MN) assay, which demonstrated superior sensitivity in detecting genotoxic potential and provided stronger evidence of mutagenic effects compared to the comet assay, are consistent with findings from studies conducted in cell cultures by Zunec et al. (2016). Similarly, Pfau et al. (1999) reported that the MN assay exhibited greater sensitivity than the comet assay in MCL5 cells exposed to heterocyclic aromatic amines. According to multiple studies, the assessment of xenobiotic genotoxicity can be effectively performed using different cell lines and is more robustly interpreted when the comet and micronucleus (MN) assays are applied in combination (Gajski et al. 2016). The integrated application of these assays provides more comprehensive and consistent insights into the toxicity and mechanisms of action of the tested compounds, as well as their potential adverse effects on living organisms (Tewari et al. 2005; Cordelli et al. 2021).

Notably, genotoxic effects were more pronounced in Association 8, where cadaverine levels were reduced while putrescine remained at the same concentration as in 6. In Association 9, where both amines were further reduced, the genotoxic response was less pronounced than in 8. These results reveal a non-monotonic dose–response relationship, where the magnitude of the genotoxic effect does not follow a linear progression with concentration (Vandenberg et al. 2012). This biphasic pattern, where damage increases from Association 9 to Association 8 but declines at the highest concentration (Association 6), likely arises from the combined effects of cadaverine and putrescine, an association previously documented to potentiate cytogenotoxicity in other bioindicators (Braga et al. 2024a, 2024b).

We hypothesize that Association 6 may be consistent with a threshold-like response, as described in oxidative stress models (Lushchak 2011), although this mechanism was not directly assessed in the present study. At this level, these concentrations (which were found to be the highest non-cytotoxic doses for Speedy cells in our preliminary assays) may be compatible with cellular responses involving antioxidant and DNA repair pathways, as previously reported for related systems (Campos-Pereira et al. 2025), although these processes were not directly measured here. This protective response mitigates the formation of micronuclei and nuclear buds (Fenech et al. 2011), whereas Association 8 showed higher genotoxic responses compared to other conditions, suggesting a non-linear pattern of response. In this intermediate state, the concentration is sufficient to induce chromosomal instability but remains below the critical trigger required for full homeostatic compensation (Lushchak 2011). This persistent risk to genomic integrity is evident even at the lower levels tested in Association 9, suggesting that genomic instability may occur even at lower tested levels and that the interaction between these biogenic amines deserves further investigation.

In this context, these observations may help to explain the differences observed between assays, although further mechanistic studies are required. The absence of detectable alterations in Association 6 may reflect compensatory cellular processes, whereas the micronuclei and buds observed in Associations 8 and 9 likely indicate chromosome loss or malsegregation that escaped the homeostatic threshold. Consequently, as the comet assay is inherently limited in detecting aneugenic events, the presence of these biomarkers suggests that the genotoxic insult may involve mechanisms of chromosomal instability that do not necessarily involve the formation of DNA strand breaks. This may partially explain the absence of detectable effects in the comet assay, regardless of the 24-h exposure period.

An alternative approach to elucidate the effects of the combined biogenic amines CAD and PUT involved evaluating the cell cycle profile and DNA fragmentation in Speedy cells using a flow cytometry assay after PI staining (Nunez 2001). In these assays, exposure to cells to the amine combinations for 24 h did not elicit detectable DNA damage. However, after 48 h, Association 5 induced DNA fragmentation consistent with apoptotic cell death.

The 48-h exposure period halted the progression of the cell cycle by arresting it in the S phase, which negatively impacted DNA replication. DNA damage from external factors can activate S-phase checkpoints, causing a slowdown or complete stop of cell cycle progression to prevent incorrect cell division (Tamborlin et al. 2020). Notably, even with an exposure duration of 48 h, the non-cytotoxic (Associations 6, 8, and 9) and genotoxic (Associations 8 and 9) combinations did not result in DNA fragmentation or cell cycle damage.

Chen et al. (2015) evaluated the effect of putrescine on cell proliferation and apoptosis in the human skin fibroblast line (HSF) following 24-h exposure. The authors verified that low concentrations of the amine (0.5, 1.0, 5.0, and 10 μg/mL) stimulated cell proliferation and decreased the apoptosis rate, whereas higher concentrations (100, 500, and 1000 μg/mL) suppressed cell proliferation and induced apoptosis in fibroblast cells. The authors observed that increasing putrescine concentrations promoted a dose-dependent increase in apoptosis levels in the HSF cell line.

Campos-Pereira et al. (2025) evaluated the genotoxic effects of putrescine in HepG2 cells exposed to this amine for 24 h. The results of the micronucleus (MN) and comet assays, performed at concentrations of 0.5, 1.4, 2.3, and 3.2 mM, revealed DNA damage as well as alterations in the expression of genes involved in oxidative stress pathways and DNA repair mechanisms. Additionally, the data suggest that the interaction of putrescine with nucleic acids may induce cytotoxic and/or neutral effects depending on the cell cycle phase. These findings are supported by two other reviews on the role of polyamines in modulating cellular function and physiology (Igarashi and Kashiwagi 2010; Schibalski et al. 2024).

Del Rio et al. (2019) evaluated the cytotoxicity of CAD (0 to 150 mM) and PUT (0 to 160 mM) diamines in HT29 intestinal cells to determine the legal limits of these diamines in food. The study revealed that the concentrations most commonly found in food were cytotoxic to the intestinal cells, as they induced necrosis in these cells. The authors attributed this toxic effect to the interaction of these amines with anionic phospholipids, resulting in the destabilization of cellular membranes.

Studies conducted by several authors on plant and animal organisms exposed to putrescine and cadaverine have revealed cytotoxic effects of these biogenic amines. Braga et al. (2024a) reported phytotoxic effects of both cadaverine and putrescine in experiments with *Lactuca sativa* and *Allium cepa*, which were mediated through aneugenic and clastogenic mechanisms. Based on these findings, the authors observed that the combined exposure to cadaverine and putrescine leads to potentiated cytotoxic and mutagenic effects. Other studies on cadaverine and putrescine, conducted in zebrafish, demonstrated embryotoxic effects resulting from the absorption of these chemical compounds through the chorion, attributed to the nucleophilic and polycationic nature of these biogenic amines (Pelka et al. 2017; Braga et al. 2024b). Unal et al. (2008) found that biogenic amines neutralize the negative charges of phosphate groups in DNA, thereby promoting the folding of the genetic material and enhancing its interaction with proteins, as well as leading to increased DNA condensation. Such interactions are considered detrimental to DNA and associated proteins due to the presence of nucleophilic sites within the DNA structure (Unal et al. 2008).

Our studies using the Speedy cell line demonstrated that a 24-h exposure to cadaverine (CAD) combined with putrescine (PUT) was genotoxic, even at lower CAD concentrations (Associations 8 and 9). After 48 h, however, only the highest concentration (Association 5) induced significant alterations in the cell cycle and DNA fragmentation, indicating extreme toxicity to Speedy cells. Collectively, these results highlight that CAD and PUT, commonly present in cemetery leachate, may indicate potential biological effects at the cellular level, underscoring the need for further studies to assess environmental risk.

The temporal divergence observed between 24 and 48 h exposures may indicate time-dependent cellular responses. In Speedy cells, early exposure (24 h) was associated with chromosomal instability, as evidenced by micronucleus formation, without detectable cytotoxicity or apoptotic DNA fragmentation. This pattern may be consistent with cellular responses that temporarily maintain viability despite genotoxic stress, as discussed in studies on biogenic amines (Campos-Pereira et al. 2025), although the underlying mechanisms were not directly investigated in the present study. At 48 h, the highest concentration (Association 5) was associated with DNA fragmentation and alterations in cell cycle progression, suggesting increased cellular stress and possible impairment of cellular homeostasis over time. Together, these observations suggest a progressive cellular response, where early genotoxic effects precede detectable cytotoxic outcomes.

The potentiation of effects previously documented in other bioindicators (Braga et al. 2024a, 2024b) underscores the importance of evaluating these amines from a mixture toxicology perspective. In our study, the non-monotonic dose–response relationship suggests that the association between CAD and PUT deviates from a simple dose addition model. This indicates a concentration-dependent interaction, where the specific ratios within the mixture may modulate cellular defenses differently than their individual components. Such deviations from additivity are common in complex mixtures, where synergistic-like interactions or threshold-dependent recovery mechanisms can significantly alter the final toxicological outcome (Vandenberg et al. 2012).

Despite these findings, some limitations of the present study should be acknowledged. First, the concentrations tested were selected based on available toxicological references and experimental design considerations, as quantitative data on the actual levels of cadaverine and putrescine in necro-leachate remain scarce (Campos-Pereira et al. 2025). Therefore, although the tested combinations provide important mechanistic insights, their direct environmental representativeness should be interpreted with caution. In this context, the use of subcytotoxic concentrations based on viability thresholds is a widely accepted approach in vitro toxicology (ISO 10993–5:2009 2009; López-García et al. 2014), as sublethal effects are recognized indicators of cellular stress and genomic instability (Fenech et al. 2011; Lushchak 2011; OECD 2014). Moreover, considering that exposure to necro-leachate is typically continuous rather than episodic, and that cadaverine and putrescine are naturally generated during decomposition processes and occur simultaneously in necro-leachate (Saba et al. 2023), these findings may provide preliminary insight into potential cellular responses to sustained low-level exposure to biogenic amines. In this sense, the environmental relevance of the present study lies not in reproducing a defined environmental concentration, which remains unknown, but in demonstrating that these decomposition-derived compounds are capable of inducing early cellular and genomic alterations even under non-cytotoxic conditions.

Second, the comet assay was performed after a 24 h exposure period, which may have limited the detection of transient primary DNA lesions that typically occur within the first hours following exposure (Brendler-Schwaab et al. 2005). Furthermore, as the comet assay is specifically designed to detect DNA strand breaks, it is inherently less sensitive to aneugenic events (Collins et al. 2023). To partially address this temporal limitation, complementary analyses were conducted using flow cytometry at 24 and 48 h, enabling the evaluation of cell cycle distribution and DNA fragmentation. While this approach does not replace early time-point comet analysis, it allowed the identification of delayed cytotoxic and apoptotic events, contributing to the interpretation of the genotoxic profile of CAD and PUT combinations.

Finally, although the Speedy cell line represents a relevant vertebrate in vitro model aligned with current principles of reduction of animal experimentation (OECD 2014, 2017), two-dimensional (2D) cell cultures cannot fully reproduce the structural and physiological complexity of whole organisms (OECD 2018). Therefore, while this model provides valuable mechanistic insights under controlled experimental conditions, extrapolation of these findings to environmental or human health risk scenarios should be approached with caution.

This study is pioneering in its use of the Speedy cell line, derived from *Xenopus tropicalis*, to assess the toxicity of chemical compounds. The results demonstrate the applicability of this experimental model for evaluating the toxic effects of different concentrations and combinations of biogenic amines CAD and PUT. Nevertheless, data on biogenic amine concentrations in necro-leachate remain limited (Braga et al. 2024a; Campos-Pereira et al. 2025).

Therefore, future studies should quantify the biogenic amines present in necro-leachate. This recommendation is supported by the observation of genotoxic effects under different combined CAD and PUT exposure conditions. Such investigations would help to elucidate the toxicity of these compounds based on their actual concentrations in necro-leachate.

These findings may raise concerns regarding potential exposure scenarios, particularly in occupational settings involving frequent environmental contact, warranting further targeted investigation before broader risk extrapolation.

## Conclusions

This study provides initial insights into the genotoxic effects of compounds with largely unknown modes of action, particularly the biogenic amines cadaverine (CAD) and putrescine (PUT). By assessing the toxicity of these contaminants, it introduces the Speedy cell line (*Xenopus tropicalis*) as a promising bioindicator for monitoring environmental stressors associated with cemetery activities. Our results suggest that the effects of CAD and PUT in Speedy cells are concentration- and time-dependent: intermediate concentrations induced genotoxic (potentially via aneugenic mechanisms) effects after 24 h, whereas the highest concentration (Association 5) led to DNA fragmentation and cell cycle arrest after 48 h. Although these findings highlight a clear toxic potential, the actual environmental impact and risks to public health should be interpreted with caution, as they depend on specific exposure contexts and real-world concentrations in necro-leachate. Consequently, the potential of these amines as environmental pollutants reinforces the importance of further investigations to fully elucidate their ecological impacts and to characterize actual exposure levels in cemetery environments.

## Data Availability

All data generated or analyzed during this study are included in the main manuscript.
